# Supplementary material for: Functional insights into the photoactive yellow protein family from homologs, multidomain proteins, and inferred pyp operons
Source: J Bacteriol. 2025 Dec 10;208(1):e00216-25. doi: 10.1128/jb.00216-25 (PMC12826048; doi:10.1128/jb.00216-25)
Supplement: Supplemental material — Supplemental methods, Fig. S1, Tables S1 to S3, and legends for Data Sets S1 to S4. [file jb.00216-25-s0005.docx]

**Supplemental Materials and Methods**

**Homolog Search, selection of PYP homologs, and multiple sequence alignment.**

The 125 amino acid PYP sequence from *H. halophila* (PDB: 1NWZ) was used as the query for a NCBI PSI-BLAST against the non-redundant database (December 2022) using the PAM250 matrix (word size 3 and gap costs 14,2) to generate a list homologs of PYP (1, 2). The resulting hits from NCBI were filtered to remove the duplicates and man-made mutant sequences and then used to generate a multiple sequence alignment (MSA). To aid in obtaining a high-quality MDA, the sequences were truncated to a length of 145 amino acids. The G-INS-i method in MAFFT version 7.490 was then used to obtain a high-quality multiple sequence alignment using the GlobalPair approach (3). Next, CIAalign version 1.0.17 was used to remove sequences containing insertions that occur in less than 50% of the sequences. This step also removed the ends of sequences if they align poorly with the majority of the alignment and removed sequences that are shorter than 50 amino acids (4). Of the 998 remaining PYP homologs, 15 did not contain the functionally essential residue Cys69. This residue is the attachment site of the *p*CA chromophore, and the C69A mutation was found to be the only mutant in a complete mutagenesis scan of Hhal PYP that eliminates function from PYP (5). Such PYP homologs lacking Cys69 have been observed previously (6, 7) and their function remains unknown. The analyses reported here were performed on 984 PYP homologs containing Cys69. To study the taxonomic distribution of PYP homologs, the NCBI database was used to successfully retrieve taxonomic information for 984 of the PYP homologs. The resulting MSA was then used to create the phylogenetic tree. IQ-TREE 1.16.12 was used to create a high quality maximum likelihood phylogenetic tree of the PYP homologs using a bootstrap value of 1000 (8, 9).

**NCBI Domain Analysis server**

To identify candidate multi-domain proteins containing a PYP domain, BLAST hits that were longer than 200 residues were retrieved, since the typical length of PYP homologs are approximately 125 residues (and the typical length of PAS domain is approximately 100 residues). The NCBI Batch web conserved domain (CD) search tool was used to identify domains in these proteins (10). The hits were screened against the Pfam (19638 PSSMs) database, with the expected value threshold kept as the default of 0.01 (11). Once complete, the data was downloaded in concise data mode, where only the superfamilies were analyzed. A possible complication of the two approaches used here to identify proteins that are functionally related to PYP (multidomain proteins and predicted *pyp* operons) is that this genetic association is a random event not indicative of a functional relation with PYP. Therefore, we only analyzed multidomain proteins and genes in *pyp* operons that were identified 3 times in 2 or more different organisms (unless otherwise stated in the text). 15 of the 984 PYP homologs initially flagged as multidomain proteins consisted of a PYP domain and a C-terminal tail, while 25 contained an N-terminal tail that did not contain a detectable conserved domain. Such a tail region in PYP was first for the PYP homolog from *Salinibacter ruber* (12). The presence and absence of predicted trans-membrane α-helices in MCP-PYP fusion protein was verified based on hydrophobicity plots.

**KEGG Operon Analysis**

The structure of predicted *pyp* operons were analyzed using the Kyto Encyclopedia of Genes and Genomes (KEGG) (13, 14). KEGG.BLAST was used to retrieve PYP homologs in the database. The amino acid sequence of Hhal PYP 1 (pdb:1nwz) was used as a query using default parameters. Once the hits were retrieved, operons containing the *pyp* gene were visually inspected for the presence of possible genes encoding annotated proteins that may be functionally related to PYP. To be considered as a gene belonging to a predicted operon, all genes in this operon were required to be transcribed in the same direction and separated by fewer than 100bp from the neighboring gene. In the work reported here, we identified and studied a total of 130 PYP operons. 17 of these were excluded from further analysis because the PYP homolog encoded did not contain the functionally essential Cys69. Genes identified as candidates for being functionally related to PYP were only discussed here if they were identified in predicted *pyp* operons more than twice.

**ColabFold: Alphafold-based multi-domain protein structure prediction**

We used ColabFold, which employs MMseq2 for a fast homology search, as well as AlphaFold2 for faster and more accurate structure prediction to examine the predicted structure of selected multi-domain proteins. The amino acid sequences of these proteins were submitted and the program was operated using default settings (15). The predicted structures were then analyzed using PyMol 3.0.4 (16).

**Construction of an *E. coli* strain overproducing the PYP domain from *N. alkalilacustris***

*Nitrincola alkalilacustris* DSM-No Strain 29817 was ordered from DSMZ. The strain was grown for 24 hours in marine broth 2216 from Millipore Sigma (cat: 76448). Once the culture was grown, genomic DNA (gDNA) was extracted using the Quick-DNA Fungal/Bacterial Miniprep Kit from Zymo Research (cat: D6005). The *pyp* gene as identified in the available genome sequence for this organism was amplified using the polymerase chain reaction (PCR) with gene specific primers and the extracted gDNA as the template. Gel electrophoresis was used to determine if the resulting PCR product had the expected size (~375 bp). The Zero Blunt TOPO PCR cloning kit from ThermoFisher Scientific (ref 451245) was used to switch the orientation of the *pyp* gene using the protocol provided by ThermoFisher. After the cloning reaction, the plasmid was transformed into NEB® 5-alpha competent *Escherichia coli* (High Efficiency) cells and plated onto Luria-Bertani (LB) agar with kanamycin (50 µg/mL) plates. Transformants were screened by partially sequencing the plasmid using the M13 forward primer.

Next, the *pyp* insert was amplified with PCR and the vector pET28b+ was linearized with NcoI (R0193T) and BamHI (R0136S) from New England Biolabs (NEB) restriction enzymes. Assembly (E5510S) from NEB was used to assemble the newly constructed plasmid, which also provides kanamycin resistance for selection. The completed plasmid was transformed into NEB® 5-alpha competent *E. coli* (High Efficiency) cells, and transformants were selected on LB+ kanamycin (50 µg/mL) plates to select for the assembled plasmid. Plasmid DNA was extracted and sent for sequencing at the DNA Protein Core Facility at Oklahoma State University, where T7 promoter primers were used to confirm the inserts sequence and integrity. The plasmid was then transformed into *E. coli* strain BL21 (DE3) (C2527H) from NEB for PYP overproduction.

**Purification of the PYP domain from *N. alkalilacustris***

A LB agar plate containing 50µg/mL of kanamycin was streaked with the BL21 *E. coli* strain containing the above construct from frozen stock and incubated at 37℃ for 16-20 hours or until colonies appeared. 2 x 1L of LB broth media with 50µg/mL kanamycin was prepared in a 2.8L flask to promote aeration. The first 1L was inoculated with multiple colonies from the agar plate to create a preculture, which was grown at 37℃ with shaking at 250 rpm for 10-12 hours. After incubation, the OD_600_ of the pre-culture was measured to estimate the cell density. The cells were then pelleted at 3750 rpm (3273 x g) for 20 minutes and resuspended in the second 1L LB flask with 50µg/mL kanamycin. Once resuspended, the initial OD_600_ of the resulting culture was measured, followed by incubation at 37℃ while shaking at 250 rpm. The OD_600_ was measured every 45 – 60 minutes until the OD_600_ had reached 0.8-1.0. The culture was then induced with 1mM (final concentration) IPTG and incubated for another 7-8 hours. After incubation, the cells were harvested at 3750 rpm (3273 x g) for 20 minutes. The supernatant was discarded, and the cell pellet was frozen at -80℃ until ready for purification.

For PYP purification, the cell pellet was thawed and the cells were lysed with 90mL/L of 8M urea in the presence of protease inhibitor (cat: 04693116001) and DNase (cat: 10104159001), both purchased from Millipore Sigma. The mixture was stirred for 1 hour at room temperature until the pellet was fully resuspended. The solution was centrifuged at 30,000 x g for 20 minutes at room temperature. The supernatant was collected, and the urea was diluted to a final concentration of 4M with 20mM Tris pH 7.5. Immediately after diluting the solution, 400µl/L of *p*-coumaric acid anhydride (prepared as described in (17)) was added while rapidly stirring. The reaction was stirred for 60 minutes at room temperature and then dialyzed overnight against 20 mM Tris pH 8.5, stored in the dark at 4℃.

The resulting protein solution was applied to a DEAE Sepharose Fast Flow (cat: 17-0709-01 purchased from Cytiva) column pre-equilibrated with 20 mM Tris pH 8.5 and washed with 20 mM Tris pH 8.5. Elution was carried out in steps with 20, 40, 60, and 80 mM NaCl in 20mM Tris pH 8.5. Fractions were collected and their absorbance was measured at 280 nm and 446 nm using an HP 8453 diode array spectrophotometer to calculate the purity index (PI) by calculating abs280/abs446. The optimal PI for WT PYP1 is 0.43. The purest fractions were dialyzed overnight in 20mM Tris pH 7.5 at 4℃. A second round of purification was performed using a Q Sepharose Fast Flow (cat:17-0510-01 from Cytiva) column pre-equilibrated with 20 mM Tris pH 7.5. The protein was loaded onto the column, washed and eluted with 20, 40, 60, and 80 mM NaCl in 20 mM Tris pH 7.5. Fractions were collected and the purest ones were dialyzed against 20 mM Bis-Tris pH 6.0 overnight at 4℃. For the final purification step, the Q Sepharose High Performance (cat: 17-1014-01 from Cytiva) column was pre-equilibrated with 20 mM Bis-Tris pH 6.0. The protein was loaded, washed, and eluted with 20, 40, 60, 80mM NaCl in 20 mM Bis-Tris pH 6.0. UV/Vis absorbance spectra of the resulting fractions were measured, and the pure protein with PI of 0.51 was dialyzed against 20 mM Tris pH 7.5 overnight at 4℃. The protein was aliquoted and stored at -80℃.

The extinction coefficient of the PYP domain from *N. alkalilacustris* was determined by measuring the absorbance spectrum before and after denaturation with 2% SDS as previously described (18). Spectroscopic data were plotted and analyzed using OriginPro (version 2023b; Origin Lab Northhampton, MA, USA) software package.

**TEM imaged of *Nitrincola alkalilacustris* DSM-No Strain 29817**

*N. alkalilacustris* was grown in marine broth for 24h at 30℃ shaking at 220 rpm. After growth the culture was pelleted and the supernatant was discarded. A fixative agent, 2% buffered glutaraldehyde at pH 7.0, was carefully added to not disturb the cell pellet and incubated at room temperature for 15 minutes. The sample was then resuspended and placed on a formvar coated grid and a drop of 2% uranyl acetate was added to negatively stain the sample. This sample was incubated at room temperature for 1 minute. Any excess liquid on the grid was removed using filter paper. Once the sample was prepared, transmission electron microscopy (TEM) images were taken using a JEOL JEM-2100 instrument at the Oklahoma State University Microscopy Laboratory.

**Motility Assay of *Nitrincola alkalilacustris* DSM-No Strain 29817**

Marine broth plates at agar concentrations of 0.5%, 0.4%, 0.3% and 0.2% were prepared to identify at which concentration *N. alkalilacustris* cells are most motile. Using an already established colony from a 1.5% agar plate, one colony was used to stab the center on the lower agar concentration plates. Once inoculated, the plates were incubated at 30℃ for up to 5 days and regularly inspected visually.

**Supplemental Figures and Tables**

**
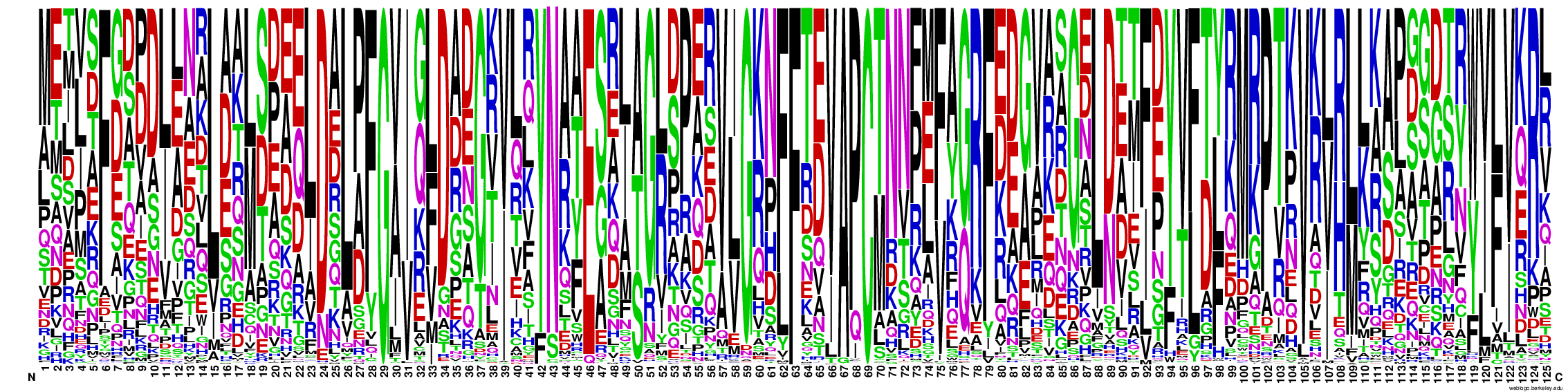
**

**Supplemental Figure S1**. Consensus sequence of PYP generated using the web server at <https://weblogo.berkeley.edu/logo.cgi>.

**Supplemental Table S1. Bioinformatics of genes components of the chemotaxis machinery in *Nitrincola alkaliacustris.***

| Protein | KEGG accession number *E.coli* K12 MG1655 | NCBI accession number *Nitrincola alkalilacustris* | Percent Covarge | Percent Identity |
| --- | --- | --- | --- | --- |
| MCP | b1421 | WP_151604061.1 | 54% | 53% |
| AER | b3072 | [WP_151705581.1](https://www.ncbi.nlm.nih.gov/protein/WP_151705581.1?report=genbank&log$=protalign&blast_rank=1&RID=023DYR41013) | 95% | 31% |
| Che R | b1884 | [WP_151704058.1](https://www.ncbi.nlm.nih.gov/protein/WP_151704058.1?report=genbank&log$=prottop&blast_rank=1&RID=023YZXG7013) | 91% | 51% |
| Che B | b1883 | [WP_151704056.1](https://www.ncbi.nlm.nih.gov/protein/WP_151704056.1?report=genbank&log$=prottop&blast_rank=1&RID=0249BM96016) | 100% | 54% |
| Che A | b1888 | [WP_151704063.1](https://www.ncbi.nlm.nih.gov/protein/WP_151704063.1?report=genbank&log$=prottop&blast_rank=1&RID=024HV4UE013) | 98% | 47% |
| Che W | b1887 | [WP_151704062.1](https://www.ncbi.nlm.nih.gov/protein/WP_151704062.1?report=genbank&log$=prottop&blast_rank=1&RID=02507WMS016) | 93% | 57% |
| Che Y | b1882 | [WP_151704064.1](https://www.ncbi.nlm.nih.gov/protein/WP_151704064.1?report=genbank&log$=prottop&blast_rank=2&RID=02F3UXJK016) | 91% | 40% |
| Che Z | b1882 | [WP_151702678.1](https://www.ncbi.nlm.nih.gov/protein/WP_151702678.1?report=genbank&log$=prottop&blast_rank=1&RID=02FMJKUK016) | 91% | 25% |
| Fli G | b1939 | [WP_151702660.1](https://www.ncbi.nlm.nih.gov/protein/WP_151702660.1?report=genbank&log$=prottop&blast_rank=1&RID=04JJCAEZ013) | 99% | 43% |
| Fli M | b1945 | No significant similarty found | | |
| Fli N | b1946 | No significant similarty found | | |
| Mot A | b1890 | No significant similarty found | | |
| Mot B | b1889 | No significant similarty found | | |

|  | **Hhal_1820** | **BB497_00790** | **AX768_29895** | **M446_3195** | **BSY238_1129** | **HC341_15830** | **K8354_02635** | **LOKO_01494** | **WP_011339422** | **Percent Similarity** |
| --- | --- | --- | --- | --- | --- | --- | --- | --- | --- | --- |
| **Hhal_1820** |  | 71.00 | 48.55 | 29.40 | 50.55 | 63.16 | 50.65 | 72.13 | 57.56 |  |
| **BB497_00790** | 58.19 |  | 49.18 | 30.15 | 48.99 | 63.07 | 51.30 | 85.93 | 56.75 |  |
| **AX768_29895** | 30.18 | 32.06 |  | 25.59 | 67.50 | 48.63 | 46.04 | 47.99 | 51.02 |  |
| **M446_3195** | 12.55 | 12.55 | 9.69 |  | 28.57 | 28.79 | 27.22 | 29.32 | 29.83 |  |
| **BSY238_1129** | 30.66 | 29.25 | 54.30 | 10.99 |  | 50.09 | 45.04 | 50.55 | 49.81 |  |
| **HC341_15830** | 49.44 | 48.67 | 32.42 | 10.47 | 31.63 |  | 50.28 | 63.07 | 58.75 |  |
| **K8354_02635** | 31.98 | 33.52 | 27.44 | 10.19 | 26.10 | 33.64 |  | 50.47 | 49.82 |  |
| **LOKO_01494** | 58.00 | 78.71 | 31.50 | 12.41 | 30.51 | 48.67 | 34.64 |  | 56.51 |  |
| **WP_011339422** | 40.77 | 41.04 | 32.47 | 12.34 | 30.56 | 42.54 | 34.13 | 42.01 |  |  |
| **Percent Identity** | | | | | | | | | |  |

**Table S2: Percent identity and similarity of Tyrosine Ammonia Lyases (TAL) found in the *pyp* operons.** The TAL with the NCBI accession number WP 011339422 has been biochemically validated to have TAL activity in *Rhodobacter sphaeroides.*

| Organism | KEGG Accession Number | Number of PYPs | Taxonomy (Class) | Single or part of Multidomain PYP |
| --- | --- | --- | --- | --- |
| Burkholderia sp. PAMC 28687 (T#: T05699) | bui:AX768_20960 | 3 | Betaproteobacteria | Single |
|  | bui:AX768_29900 |  |  | Single |
|  | bui:AX768_29920 |  |  | Single |
| Burkholderia sp. PAMC 26561 (T#: T05481) | bum:AXG89_24860 | 3 | Betaproteobacteria | Single |
|  | bum:AXG89_27835 |  |  | Single |
|  | bum:AXG89_39425 |  |  | Single |
| Caballeronia sp. SBC2 (T#: T06459) | caba:SBC2_75410 | 5 | Betaproteobacteria | Single |
|  | caba:SBC2_75450 |  |  | Single |
|  | caba:SBC2_76250 |  |  | Single |
|  | caba:SBC2_77530 |  |  | Single |
|  | caba:SBC2_77570 |  |  | Single |
| Fibrella sp. ES10-3-2-2 (T#: T04805) | fib:A6C57_15215 | 2 | Cytophagia | Single |
|  | fib:A6C57_15220 |  |  | Single |
| Gemmatimonas groenlandica TET16 (T#: T07783) | ggr:HKW67_00425 | 2 | Gemmatimonadia | Single |
|  | ggr:HKW67_19100 |  |  | Single |
| Gemmatimonas phototrophica AP64 (T#: T04580) | gph:GEMMAAP_05245 | 2 | Gemmatimonadia | Single |
|  | gph:GEMMAAP_14260 |  |  | Single |
| Halorhodospira halophila SL1 (T#: T00462) | hha:Hhal_1818 | 2 | Gammaproteobacteria | Single |
|  | hha:Hhal_1333 |  |  | Single |
| Lichenicola cladoniae PAMC 26569 (T#: T07054) | lck:HN018_17100 | 2 | Alphaproteobacteria | Single |
|  | lck:HN018_19360 |  |  | Single |
| Massilia sp. WG5 (T#: T04122) | masw:AM586_12230 | 2 | Betaproteobacteria | Single |
|  | masw:AM586_20730 |  |  | Single |
| Persicimonas caeni YN101 (T#: T06769) | pcay:FRD00_10655 | 2 | Deltaproteobacteria | Multidomain |
|  | pcay:FRD00_25285 |  |  | Multidomain |
| Salinisphaera sp. LB1 (T#: T05476) | saln:SALB1_2011 | 2 | Gammaproteobacteria | Single |
|  | saln:SALB1_1331 |  |  | Single |
| Skermanella sp. TT6 (T#: T07020) | skt:IGS68_00180 | 2 | Alphaproteobacteria | Single |
|  | skt:IGS68_20610 |  |  | Multidomain |
| Spirosoma pollinicola HA7 (T#: T05236) | spir:CWM47_00440 | 3 | Cytophagia | Single |
|  | spir:CWM47_16640 |  |  | Single |
|  | spir:CWM47_24145 |  |  | Single |
| Skermanella rosea KEMB 2255-458 (T#: T07666) | sroe:JL101_000185 | 2 | Alphaproteobacteria | Single |
|  | sroe:JL101_005395 |  |  | Multidomain |
| Stigmatella aurantiaca DW4/3-1 (T#: T01425) | sur:STAUR_3324 | 2 | Myxococcia | Single |
|  | sur:STAUR_6414 |  |  | Single |

**Supplemental Table S3: Description of organisms that encode more than one PYP in their genome.**

**Legends for Supplemental Excel files:**

**Legend for file “MultidomainPYP_Data”.** Analysis of domain structure of 168 multi-domain PYP homologs. The conserved domains in each protein are listed, with the PYP domain indicated in yellow.

**Legend for file ‘Operon_Data”.** Analysis of the predicted operon structure of 130 *pyp* genes. For each predicted operon gene annotations are provided, together with information on the length of the predicted proteins, the length of the intergenic regions and the direction of transcription. Recurring proteins are color coded to aid in visual analysis of the data.

**Legend for file ‘PYPAlignment”.** Amino acid sequences of 986 PYP homologs, with their corresponding gene names and organism of origin.

**Legend for file ‘PYPTaxonomy”.** List of 618 bacteria containing a PYP homolog with their TaxID and information about their taxonomy. The listed protein accession numbers are for the PYP homologs in these genomes.

References

1. Altschul SF, Gish W, Miller W, Myers EW, Lipman DJ. 1990. Basic local alignment search tool. J Mol Biol 215:403–410.

2. Altschul SF, Madden TL, Schäffer AA, Zhang J, Zhang Z, Miller W, Lipman DJ. 1997. Gapped BLAST and PSI-BLAST: a new generation of protein database search programs. Nucleic Acids Res 25:3389–3402.

3. Katoh K, Misawa K, Kuma K, Miyata T. 2002. MAFFT: a novel method for rapid multiple sequence alignment based on fast Fourier transform. Nucleic Acids Research 30:3059–3066.

4. Tumescheit C, Firth AE, Brown K. 2022. CIAlign: A highly customisable command line tool to clean, interpret and visualise multiple sequence alignments. PeerJ 10:e12983.

5. Philip AF, Kumauchi M, Hoff WD. 2010. Robustness and evolvability in the functional anatomy of a PER-ARNT-SIM (PAS) domain. Proceedings of the National Academy of Sciences 107:17986–17991.

6. Meyer TE, Kyndt JA, Memmi S, Moser T, Colón-Acevedo B, Devreese B, Van Beeumen JJ. 2012. The growing family of photoactive yellow proteins and their presumed functional roles. Photochem Photobiol Sci 11:1495–1514.

7. Xing Jiawei, Gumerov Vadim M., Zhulin Igor B. 2022. Photoactive Yellow Protein Represents a Distinct, Evolutionarily Novel Family of PAS Domains. Journal of Bacteriology 204:e00300-22.

8. Hoang DT, Chernomor O, von Haeseler A, Minh BQ, Vinh LS. 2018. UFBoot2: Improving the Ultrafast Bootstrap Approximation. Molecular Biology and Evolution 35:518–522.

9. Nguyen L-T, Schmidt HA, von Haeseler A, Minh BQ. 2015. IQ-TREE: A Fast and Effective Stochastic Algorithm for Estimating Maximum-Likelihood Phylogenies. Molecular Biology and Evolution 32:268–274.

10. Wang J, Chitsaz F, Derbyshire MK, Gonzales NR, Gwadz M, Lu S, Marchler GH, Song JS, Thanki N, Yamashita RA, Yang M, Zhang D, Zheng C, Lanczycki CJ, Marchler-Bauer A. 2023. The conserved domain database in 2023. Nucleic Acids Res 51:D384–D388.

11. Finn RD, Bateman A, Clements J, Coggill P, Eberhardt RY, Eddy SR, Heger A, Hetherington K, Holm L, Mistry J, Sonnhammer ELL, Tate J, Punta M. 2014. Pfam: the protein families database. Nucleic Acids Res 42:D222-230.

12. Memmi S, Kyndt J, Meyer T, Devreese B, Cusanovich M, Van Beeumen J. 2008. Photoactive yellow protein from the halophilic bacterium Salinibacter ruber. Biochemistry 47:2014–2024.

13. Kanehisa M, Furumichi M, Tanabe M, Sato Y, Morishima K. 2017. KEGG: new perspectives on genomes, pathways, diseases and drugs. Nucleic Acids Res 45:D353–D361.

14. Kanehisa M, Goto S. 2000. KEGG: kyoto encyclopedia of genes and genomes. Nucleic Acids Res 28:27–30.

15. Mirdita M, Schütze K, Moriwaki Y, Heo L, Ovchinnikov S, Steinegger M. 2022. ColabFold: making protein folding accessible to all. Nature Methods 19:679–682.

16. Schrödinger, LLC. 2015. The PyMOL Molecular Graphics System, Version 3.0.

17. Imamoto Y, Ito T, Kataoka M, Tokunaga F. 1995. Reconstitution photoactive yellow protein from apoprotein and p-coumaric acid derivatives. FEBS Letters 374:157–160.

18. Imamoto Y, Koshimizu H, Mihara K, Hisatomi O, Mizukami T, Tsujimoto K, Kataoka M, Tokunaga F. 2001. Roles of amino acid residues near the chromophore of photoactive yellow protein. Biochemistry 40:4679–4685.
